# Inhibition of N6-Methyladenosine Accumulation by Targeting METTL3 Mitigates Tau Pathology and Cognitive Decline in Alzheimer’s Disease

**DOI:** 10.21203/rs.3.rs-8379573/v1

**Published:** 2025-12-19

**Authors:** Lulu Jiang, Allison Tucker, Camron Sepehri, Dev Patel, Qingbo Wang, Shuo Yuan, Eliana Sherman, Yicheng Chen, Joey Beh, Addysen Downey, Daniel Goldberg, Weronika Gniadzik, Xin Ma

**Affiliations:** University of Virginia; University of Virginia; University of Virginia; University of Virginia; University of Virginia; University of Virginia; University of Virginia; University of Virginia; University of Virginia; University of Virginia; University of Virginia; University of Virginia; University of Virginia

**Keywords:** Alzheimer’s disease, tauopathy, N6-methyladenosine, RNA modification, cognition, METTL3 inhibitor, STM2457

## Abstract

Dysregulation of N6-methyladenosine (m6A) modification of RNA has emerged as a novel feature of Alzheimer’s disease (AD). Here, we investigate the relationship between m6A modification and AD pathology, and the therapeutic potential of modulating excessive m6A via its “writer” methyltransferase METTL3 in a humanized P301S tau transgenic mouse model of AD (PS19). We observed significantly elevated m6A levels in human post-mortem AD frontal cortex tissue compared to healthy controls, which positively correlated with hyperphosphorylated tau and amyloid-β (Aβ) deposition. These effects were recapitulated in the PS19 tau mice model of AD. Importantly, treatment of PS19 mice with the METTL3 inhibitor STM2457 reduced excessive m6A, alleviated tau pathology, and attenuated neurodegeneration. Behavioral assessments further demonstrated that STM2457-treated PS19 mice exhibited significantly improved learning and memory relative to untreated PS19 mice. Our results identify m6A as a critical contributor to AD pathogenesis and demonstrate that pharmacological inhibition of METTL3 represents a promising therapeutic strategy to improve cognition in AD.

## Introduction

In the last 20 years, reported deaths from Alzheimer’s disease (AD) have increased over 140%, emphasizing the importance of early detection and new disease-modifying treatment strategies^1^. For decades, AD research has been focused on identifying hallmarks of amyloid-β (Aβ) deposition and aggregation of tau neurofibrillary tangles. Emerging research on the role of RNA modifications in AD pathogenesis is a promising research direction for elucidating AD beyond proteiopathies^2–4^. N6-methyladenosine (m^6^A) RNA modification is a key regulator of RNA metabolism and has been studied extensively in the context of neurodevelopment and oncogenesis, though its role in neurodegeneration remains underexplored and debated. Important roles of m^6^A in RNA metabolism include pre-mRNA processing, splicing, nuclear export, mRNA expression, and RNA degradation^5–14^. Dysregulation of m^6^A machinery, including its writers, readers, and erasers, has been linked to impaired memory formation and cognitive decline^5,15–25^. METTL3 is a core writer protein and catalytic portion of the METTL3-METTL14 methyltransferase complex, which carries out the modification resulting in m^6^A RNA^26–28^. Elevated expression of METTL3 has been implicated in AD and related rodent models^29^. Further investigation into m^6^A’s impact on key AD-related pathways could provide novel therapeutic targets, offering a fresh perspective on disease intervention and potential RNA-based treatments.

Our previous research discovered a novel association of m^6^A RNA with oligomeric tau (oTau) in the neuronal cytoplasm through the HNRNPA2B1 RNA binding protein, which behaves as an m^6^A reader. Our finding of a 3–5-fold increase of cytoplasmic m^6^A abundance in disease tissues (P301S tau mice and AD-affected human brain tissue) suggests that m^6^A plays a significant role in the pathogenic pathway of AD. This m^6^A-HNRNPA2B1-oTau interaction contributes to oTau’s integrated stress response and reduced protein synthesis^30^. Given the central role of hyperphosphorylated and oligomeric tau in AD pathology, these findings highlight the potential for modulating excessive m^6^A accumulation as a strategy to control tau aggregation.

To comprehensively assess m^6^A RNA dynamics during AD progression and aging, we analyzed the correlation between m^6^A RNA accumulation and pathological Aβ and tau deposition in human post-mortem brain tissue as well as in mouse models of tauopathy across different ages. We found a consistent increase in m^6^A RNA levels that strongly correlated with the accumulation of pathological tau and Aβ in human brains from early to late Braak stages, which was further confirmed in aging and disease-progression of mouse models. To explore the therapeutic potential of targeting excessive m^6^A RNA accumulation, we examined the effects of STM2457, a selective METTL3 inhibitor, on AD-related pathology in P301S PS19 tauopathy mice. Remarkably, STM2457 treatment rescued cognitive function, reduced pathological tau accumulation, prevented neuronal loss, and ameliorated neuroinflammation, as demonstrated by behavioral assessments, immunostaining, and biochemical analyses.

## Results

### Characterization of m^6^A modifications associated with tau hyperphosphorylation and Aβ deposition across Alzheimer’s disease progression

**Figure 1:**
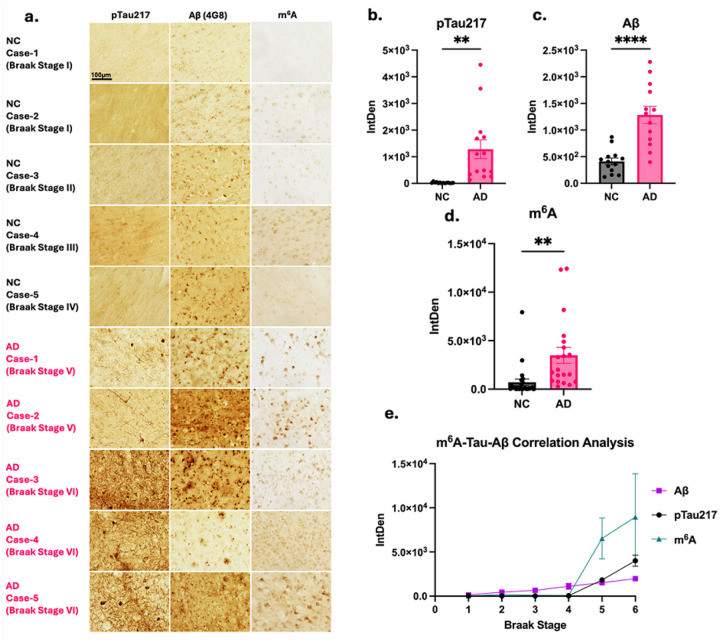
Correlation between pathological progress of tau hyperphosphorylation, Aβ plaques deposition, and excessive m^6^A accumulation. **(a)**, Representative images of 3,3’-Diaminobenzidine (DAB) staining of tau (pTau217 antibody) and Aβ (4G8 antibody) pathology in parallel with m^6^A labeling in post-mortem brain tissue of cognitively normal control (NC) and AD brain sections of Brodmann’s area 10. Scale bar = 100μm. **(b)**, Quantification of pTau217 intensity by integrated density (IntDen) in post-mortem brain tissue. **(c)**, Quantification of Aβ plaques (4G8 antibody) intensity by IntDen in post-mortem brain. **(d)**, Quantification of m^6^A intensity by IntDen in post-mortem brain tissue. **(e)**, Correlation analysis demonstrating relationship between pTau217, Aβ, and m^6^A pixels per section across Braak stages. The Pearson correlation coefficient between m^6^A and tau pathology was r = 0.97 (*p*=0.001), correlation coefficient with Aβ was r = 0.89 (*p* = 0.018). **p < 0.01, ***p < 0.005, ****p < 0.001. Error bars = SEM. Statistical differences were analyzed using unpaired Student’s t-test. *N* = 8 brains per condition. *n* = 16 sections per condition **(b-e)**.

Previous investigations examining m^6^A RNA levels in human post-mortem brain tissue from AD individuals have yielded conflicting results. Work examining the anterior cingulate gyrus and middle temporal gyrus found reduced levels of m^6^A RNA^19,31^, however, when the anterior frontal gyrus was examined, there were elevated levels of m^6^A^30^. Thus, we sought to reaffirm this cortical increase in m^6^A RNA in post-mortem brain tissue. We examined post-mortem brain tissue collected from Brodmann area 10; this region was selected based on consistent evidence from prior studies indicating a strong positive correlation between altered gene expression and disease severity^32^. The brain tissue in this study was collected from eight AD patients and eight cognitively normal control (NC) individuals. The results shown include brain tissue from NC individuals between Braak stages I-IV and AD individuals between Braak stages V-VI. Tau phosphorylated at threonine 217 (pTau217) is an early AD marker that correlates with disease severity and can be used to evaluate pathogenic tau in tissue^33,34^. Using 3,3’-Diaminobenzidine (DAB) staining, pTau217, Aβ (4G8 antibody), and m^6^A RNA levels were evaluated (Fig. 1a). As expected, we observed elevated pTau217 and Aβ pathology in AD brains (Fig. 1a–c). When comparing AD to NC, there was significantly more m^6^A in the AD post-mortem brains than the controls (Fig. 1a, d). Additionally, pTau217, Aβ, and m^6^A were all more prominent in later Braak Stages (V and VI) (Fig. 1e). Correlation analysis between pTau217, Aβ, and m^6^A revealed that pathologies were positively correlated with increased levels of m^6^A. The Pearson correlation coefficient (R) between m^6^A and tau pathology was 1.00 (*p*=2.21×10^−5^), while the correlation coefficient with Aβ was 0.80 (*p*=0.055) (Fig. 1e). This data reaffirms the presence of elevated m^6^A levels in cortical regions of AD post-mortem brain tissue, in alignment with the pathological accumulation of Aβ and tau and shows a particularly strong correlation with pathogenic tau.

### Age-related accumulation of m^6^A in humanized P301S tau transgenic mouse model of AD

**Figure 2:**
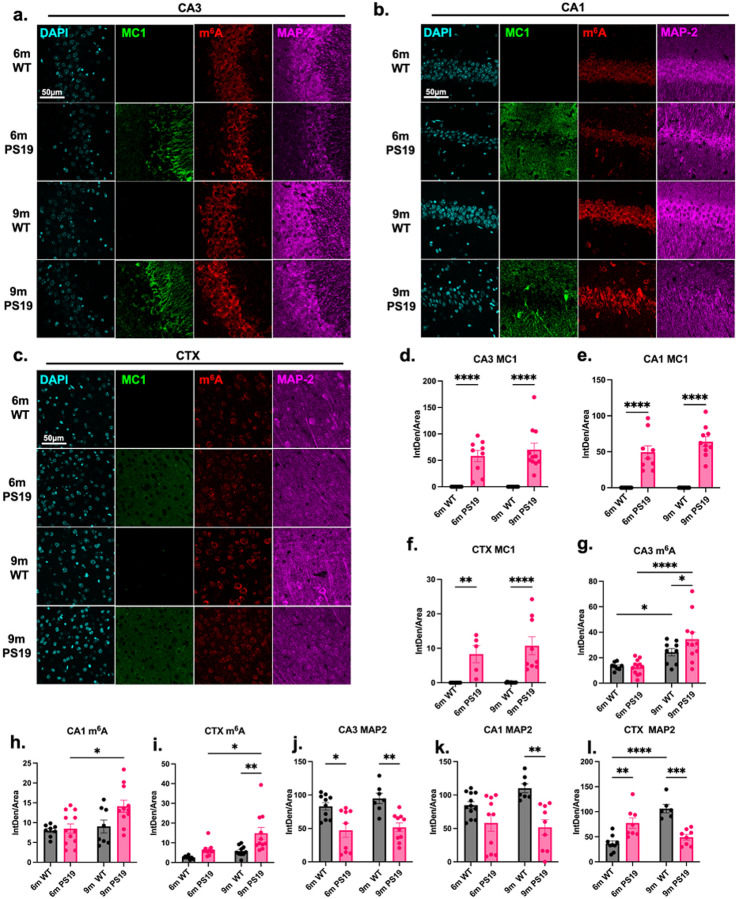
Age-related accumulation of m^6^A in humanized tauopathy mouse model of AD. **(a-c)**, Images showing DAPI (cyan), m^6^A (red), MC1 positive misfolded tau (green), and MAP2 (magenta) immunofluorescence staining in **(a)**, CA3, **(b)**, CA1, **(c)**, and cortex (CTX), regions of P301S tau (PS19) transgenic mice and C57BL/6 (WT) mice at 6-months and 9-months of age. Scale bar = 50μm. **(d-f)**, Quantification of MC1 immunofluorescence intensity by integrated density (IntDen) normalized to area in **(d)**, CA3, **(e)**, CA1, and **(f)**, CTX. **g-i**, Quantification of m^6^A immunofluorescence intensity by IntDen normalized to area as shown in **(g)**, CA3, **(h)**, CA1, and **(i)**, CTX. **(j-l)**, Quantification of neurodegeneration by MAP2 immunofluorescent intensity by IntDen normalized to area as shown in **(j)**, CA3, **(k)**, CA1, and **(l)**, CTX. Statistical analysis was performed using two-way ANOVA with Tukey’s multiple comparisons test. n. Error bars = SEM. *p<0.05, **p<0.01, ***p<0.005, ****p<0.001. *N* = 5 brains per condition. *n* = 10 sections per condition.

Previous work demonstrated an important role for m^6^A RNA methylation in regulating neurodevelopment and aging^5,20,35,36^. Data in Fig. 1 from post-mortem AD brain tissue showed that marked accumulation of m^6^A is highly correlated with tau pathology. Next, we quantified how m^6^A levels change with age in a mouse model of progressive tau pathology. To do this, we utilized PS19 mice overexpressing human P301S mutant tau and examined brain tissue collected from 6- or 9-month-old mice. To confirm the pathological relevance of the PS19 model, we labeled hippocampal and cortical regions using the MC1 antibody to identify misfolded tau. MC1 fluorescence intensity was significantly increased in PS19 compared to C57BL/6 wild-type (WT) mice in the hippocampus and cortex (CTX) at both 6- and 9-months of age (Fig. 2a–f). Considering advanced tau pathology in AD is associated with robust neurodegeneration, we also probed for MAP2, a marker of neuronal cell bodies and dendrites, to evaluate neuronal integrity. MAP2 fluorescence intensity was significantly decreased in the hippocampus and CTX of PS19 mice in 9-month brains (Fig. 2a–c and j–l). These results indicate that PS19 mice exhibit both increased tau pathology and neurodegeneration as anticipated. More importantly, staining for m^6^A revealed elevated m^6^A RNA levels in the cortex and hippocampal CA3 region of PS19 mouse brains when compared to WT controls at 9-months of age (Fig. 2a–c and g–i). Interestingly, we observed that m^6^A RNA was significantly increased in 9-month-old PS19 mouse hippocampus (CA1 and CA3) and CTX when compared to the 6-month-old PS19 group (Fig. 2g–i). However, for all brain regions examined, m^6^A levels were comparable at 6 months of age between PS19 and WT mice (Fig. 2g–i). Additionally, DAB staining of brain tissue from 5-month- and 9-month-old WT and PS19 mice revealed that m^6^A levels were significantly elevated in 9-month-old PS19 compared to age-matched WT samples, and there is a significant increase in m^6^A from the 6-month to 9-month timepoint for PS19 mice **(Extended Data Fig. 1)**. Altogether, our data suggests that the age-related increase in tau pathology and neurodegeneration in PS19 mice is accompanied by a parallel increase in m^6^A RNA modification.

### Inhibition of METTL3 reduces excessive m^6^A RNA levels in tauopathy mice

**Figure 3:**
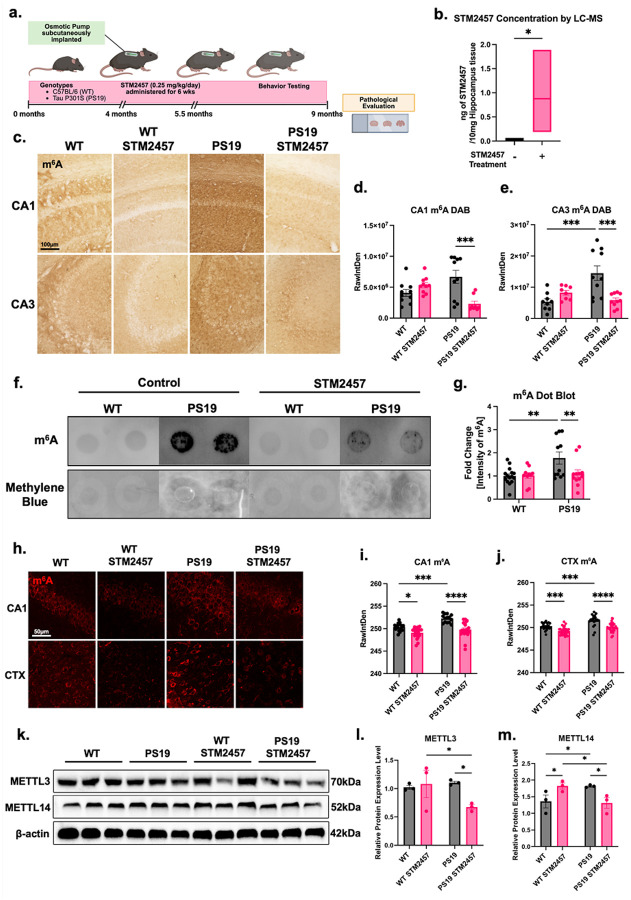
Inhibition of METTL3 reduces excessive m^6^A in mice of tauopathy. **(a)**, Schematic of timeline of STM2457 treatment in C57BL/6 mice (WT) and P301S tau transgenic mice (PS19). **(b)**, Quantification of STM2457 in 10mg hippocampal tissue from WT mice with and without STM2457 treatment by LC-MS. **(c)**, Images showing DAB staining of m^6^A in the CA1 and CA3 regions of 9-month-old WT and PS19 mice with and without STM2457 treatment. Scale bar = 100μm. **d**, **e**, Quantification of intensity of DAB stained m^6^A by raw integrated density (RawIntDen) in (**d**, CA3 and **(e)**, CA1. *N* = 5 brains per condition, *n* = 10 sections per condition. **(f)**, m^6^A RNA dot blot for PS19 and WT mice with or without STM2457 treatment. **(g)**, Quantification of m^6^A RNA dot blot signals, expressed as fold changes in m^6^A intensity (RawIntDen), in PS19 and WT mice with or without STM2457 treatment. *N* = 12 brains per condition. **(h)**, Immunofluorescence analysis of m^6^A in CA1 and CTX regions of PS19 and WT mice with or without STM2457 treatment. Scale bar = 50μm. **i**, **j**, Quantification of m^6^A immunofluorescence intensity by RawIntDen in the **(i)**, CA1 and **(j)**, CTX. **(k)**, Western blot of PS19 and WT mouse CTX probed for METTL3, METTL14, and β-actin control. **l**, **m**, Quantification of relative protein expression level of **(l)**, METTL3 and **(m)**, METTL14. *p<0.05, **p<0.01, ***p<0.005, ****p<0.001. *N* = 5 brains per condition, *n* = 10 sections per condition **(d-j)**. *N* = 3 **(k-m)**. Error bars = SEM. Statistical analysis was done using two-way ANOVA with Tukey’s multiple comparisons test **(d, e, i, j)**. Statistical analysis was done using two-way ANOVA with Fisher’s LSD **(g, l, m)**.

Our results demonstrated that m^6^A levels were elevated in both AD post-mortem brains and tauopathic mouse brains. RNA methylation is a reversible epigenetic modification, which makes it an attractive target for pharmacological intervention. Therefore, we attempted to restore the homeostasis of RNA methylation through pharmacologically removing excessive m^6^A by inhibiting its methyltransferase METTL3. STM2457 is an inhibitor of METTL3 that has been examined as a potential therapeutic for myeloid leukemia; that work demonstrated STM2457 can penetrate the blood brain barrier and infiltrate the brain^37^. STM2457 inhibits METTL3 by binding to its S-adenosyl methionine binding site, thus disrupting its catalytic activity without influencing the activity of other RNA methyltransferases^37^. Given its selective METTL3 inhibition, robust brain permeability, and high potency in IC_50_ value of 16.9 nM^37^, we utilized STM2457 as a treatment strategy, anticipating that the achievable brain dose would fall within its therapeutically effective range. STM2457 was administered continuously for six weeks at a dose of 0.25 mg/kg/day using an osmotic pump that was subcutaneously implanted in the mice starting at 4 months of age (Fig. 3a). The concentrations of STM2457 in the hippocampus of 9-month-old STM2457-treated mice were measured by liquid chromatography–mass spectrometry (LC–MS). Our result showed an average of 0.96 ng of STM2457 was detected per 10 mg of hippocampal tissue used for analysis (Fig. 3b).

Next, the efficiency of STM2457 treatment on levels of m^6^A in select brain regions was evaluated. Hippocampal levels of m^6^A in PS19 and WT mice with and without STM2457 treatment were assessed with DAB staining with m^6^A antigen probed by an m^6^A-specific antibody (Fig. 3c). Notably, PS19 mice exhibited a significant increase in m^6^A intensity in CA3 compared to WT mice. Importantly, STM2457 treatment robustly countered the presence of this excessive m^6^A (Fig. 3d). A similar pattern was observed in CA1, with STM2457 resulting in a significant reduction of m^6^A in PS19 mice. However, high levels of m^6^A were not apparent in the CA1 of untreated PS19 mice (Fig. 3e). This consistent reduction of m^6^A levels across both CA3 and CA1 indicates that STM2457 treatment effectively controls hippocampal m^6^A in PS19 mice. Changes in m^6^A levels by STM2457 were also validated through immunohistochemical analysis. We conducted an m^6^A dot blot of RNA extracted from cortical tissue of 9-month-old PS19 and WT mice and found that STM2457 treatment significantly reduced total m^6^A RNA levels in PS19 mice, but not in WT mice (Fig. 3f, g). Immunofluorescence labeling of brain tissue for m^6^A also revealed a significant reduction of m^6^A in CA1 and CTX of PS19 STM2457-treated mice (Fig. 3h–j). Together, this data demonstrates that STM2457 counteracts excessive m^6^A RNA modification in the cortex and hippocampus as measured with multiple modalities.

To further evaluate the inhibition effect of STM2457 on the expression of main methyltransferase and demethylase in the m^6^A regulatory complex, we examined the protein levels of both METTL3, METTL14, and ALKBH5 in the CTX of mice with and without treatment by western blot. Our results revealed that STM2457 treatment in PS19 mice resulted in decreased levels of METTL3 and METTL14 (Fig. 3k–m). On its own, METTL14 is a separate methyltransferase, however when METTL14 forms a complex with METTL3, METTL14 becomes catalytically inactive due to an occluded active site, and instead acts as structural support for METTL3, promoting the catalytic activity of METTL3^38^. Interestingly, METTL3 levels were not significantly altered by STM2457 treatment in WT mice, while METTL14 levels are significantly increased (Fig. 3l, m). Similarly, the demethylase ALKBH5 levels were significantly downregulated in PS19 treated mice but not WT **(Extended Data Fig. 2a, b)**. These results indicate a coordinated and complementary regulatory relationship between m^6^A writers and erasers in restoring m^6^A homeostasis.

Altogether, the evaluation on the treatment efficacy indicates that STM2457, delivered at a constant and controlled dose via osmotic pump, effectively inhibits METTL3 and reduces excessive m^6^A methylated RNA levels in the hippocampus and cortex of PS19 mice.

### STM2457-mediated reduction of m^6^A RNA rescues behavioral deficits in mouse tauopathy model.

RNA modification via m^6^A methylation has been implicated in homeostatic learning and memory processes and neurodegeneration-related deficits, along with the associated m^6^A writers and erasers^21–25,39^. The therapeutic potential of STM2457 relies heavily on its ability to prevent cognitive deficits, as these are some of the most debilitating AD sequelae. Therefore, we evaluated the effect of sustained STM2457 treatment on locomotion and cognitive behaviors of mice using a series of behavioral assays, including the Morris water maze (MWM), elevated plus maze (EPM), Y maze and open field test.

**Figure 4:**
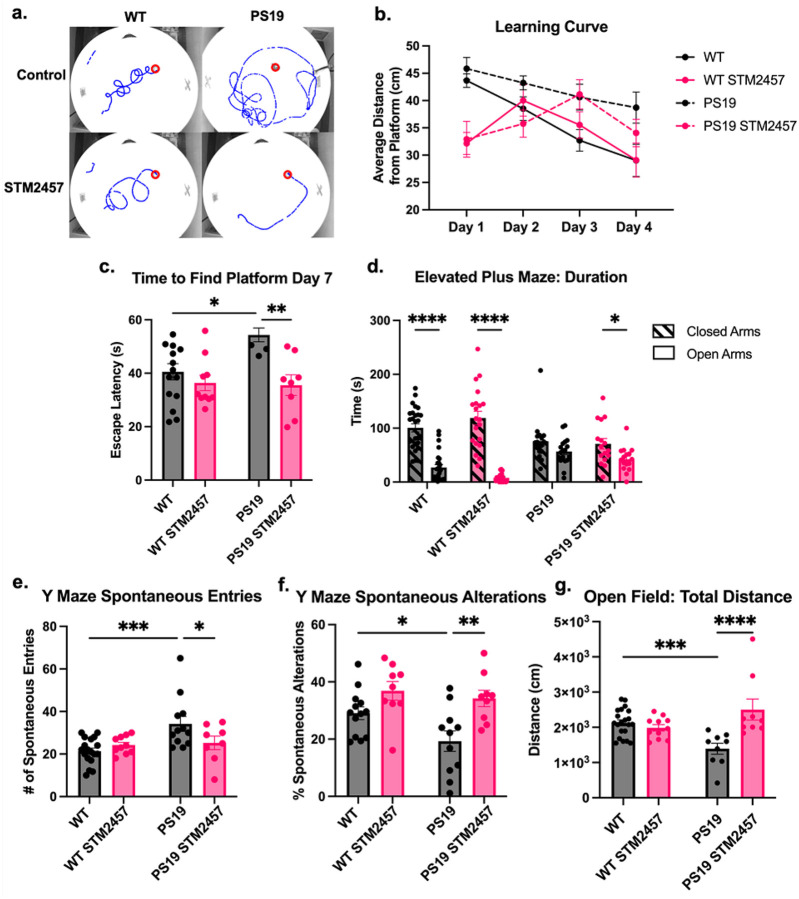
Treatment of PS19 mice with STM2457 ameliorates behavioral deficits. **(a)**, Traces from video recordings of mouse movement in the MWM on Day 7. Platform indicated with red circle. **(b)**, Average distance from platform (cm) represents mean distance from the platform in each quadrant across days 1 to 4 for PS19 and WT mice, with or without STM2457 treatment. **(c)**, Escape latency (s) indicates time spent finding the platform on Day 7. **(d)**, Time (s) spent in the open and closed arms of the EPM. **(e)**, Number of spontaneous entries into the non-starting arms of the Y maze. **(f)**, Percent spontaneous alteration of mice in the Y maze. **(g)**, Total distance (cm) traveled in the OF test. *p<0.05, **p<0.01, ***p<0.005, ****p<0.001. *N*= 10 mice per condition, *n* = 4 trials per mouse **(a-c)** and *N* = 10 mice per condition **(d-g)**. Error bars = SEM. Statistical analysis was performed using Fisher’s LSD **(b, d, e-g)**. Statistical analysis was done using two-way ANOVA with Tukey’s multiple comparisons test **(c)**.

MWM was used to evaluate spatial learning and memory. Spatial learning was assessed over four consecutive days of training by measuring the time taken to locate a hidden platform. Reference memory was evaluated over two subsequent probe trials in which the platform was removed, and preference for the former platform location was measured. On the final test day (Day 7), escape latency and search patterns were analyzed to determine the degree to which mice relied on spatial versus non-spatial strategies^40^. MWM analysis showed that 9-month-old PS19 mice exhibited significant learning and memory deficits compared to WT controls, and that these deficits were dramatically ameliorated in the same cohort treated with STM2457 (Fig. 4a–c). Specifically, PS19 mice traveled greater distances to reach the platform during the 4 days of spatial learning phase compared to WT control (Fig. 4a, b). Additionally, untreated PS19 mice took significantly longer to reach the platform on the final day (Day 7). (Fig. 4c). These results indicate impaired spatial learning and memory in PS19 mice. Treatment with STM2457 improved performance, with PS19 mice showing comparable average distance from the platform on the fourth day of training and spending similar time to reach the platform on Day-7 as WT mice (Fig. 4a–c). Notably, STM2457-treated mice exhibited a temporary increase in escape latency on days 2 to 3, coinciding with the transition from non-spatial to spatial search strategies. Following this transition, they achieved significantly lower escape latencies and shorter distances to the platform, indicating enhanced spatial memory consolidation rather than impairment. This pattern is consistent with prior studies showing that mice with superior learning performance often exhibit an initial exploratory phase characterized by increased path length or escape latency before adopting a more efficient spatial navigation strategy in the Morris water maze^41,42^.

The EPM is typically used to assess anxiety-like behaviors, however we utilized the test to evaluate cognition. Previous work demonstrated that the EPM can reveal whether the mice recognize the danger associated with the open arms, a behavior that is impaired in mice with cognitive deficits.^43^. Our EPM analysis revealed that PS19 mice exhibited impaired cognitive capacity (Fig. 4d). At baseline, WT mice explored both open and closed arms but showed a clear preference for the closed arm, reflecting their ability to recognize the risks of the open arm. In contrast, untreated PS19 mice spent similar amounts of time in both arms, indicating impaired risk recognition. STM2457-treated PS19 mice, however, demonstrated a preference for the closed arm, exhibiting a behavioral pattern similar to WT mice (Fig. 4d).

Evaluation of spatial working memory using the Y maze revealed that the number of spontaneous entries was significantly elevated in PS19 mice when compared to WT, but with treatment this difference was ameliorated (Fig. 4e). Additionally, the percent spontaneous alteration, a measure that gives insight into working memory capabilities, revealed a deficit in percent spontaneous alteration in untreated PS19 mice relative to untreated WT mice (Fig. 4f). With STM2457 treatment there was a significant increase in percent spontaneous alteration relative to untreated PS19 mice, indicative of improved spatial working memory (Fig. 4f).

The open field test was used to evaluate locomotor activity and gain insight into motor deficits commonly observed in PS19 mice. These deficits are attributed to the clasping and limb retraction that precede hind limb paralysis that develops at aged time-points with substantial pathological tau burden^44^. In the open field test, PS19 mice traveled significantly less total distance than WT mice, whereas STM2457-treated PS19 mice showed locomotor activity comparable to WT controls (Fig. 4g). This improvement indicates that PS19 mice with treatment experienced fewer motor deficits or peripheral symptoms typically preceding hind limb weakness and paralysis.

Taken together, these behavioral assays demonstrate that STM2457 treatment markedly improves learning, memory, and overall cognition in PS19 mice, which typically exhibit deficits in these behavioral paradigms.

### STM2457 treatment mitigates tau pathology and protects against neurodegeneration.

**Figure 5:**
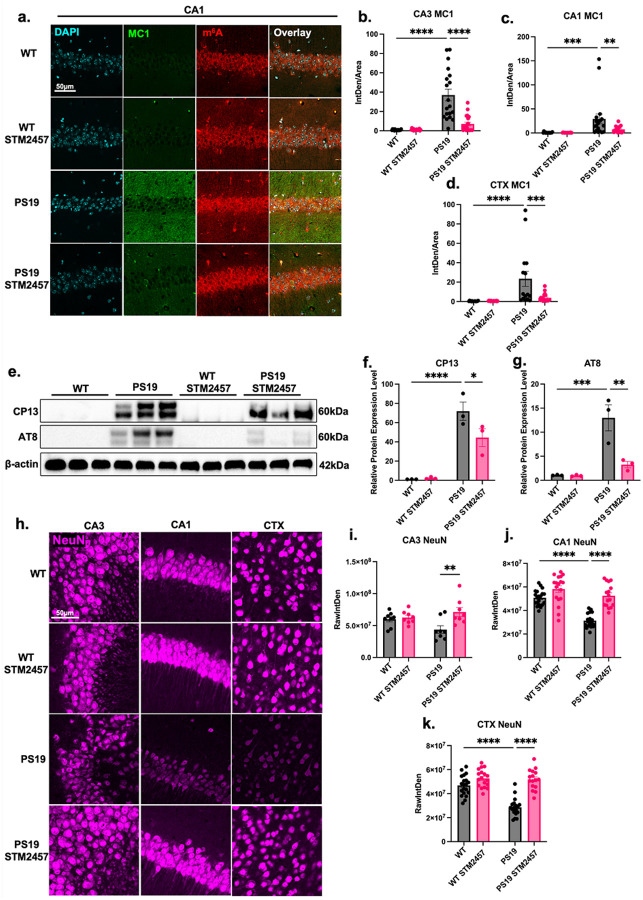
STM2457 treatment mitigates tau pathology and neurodegeneration. **(a)**, Representative immunofluorescence images of CA1 of WT and PS19 control and STM2457 treated mice with DAPI (cyan), misfolded tau (green, MC1 antibody), and m^6^A (red) Scale bar = 50μm. **b-d**, Quantification of immunofluorescence intensity of misfolded tau by integrated density (IntDen) normalized to area in the **(b)**, CA3, **(c)**, CA1, and **(d)**, CTX. *N* = 10 mice per condition, *n* = 20 sections per condition. **(e)**, Western blot analysis of phosphorylated tau at Ser202 and Ser202/Thr205, detected using CP13 and AT8 antibodies, respectively, with β-actin as the internal loading control. *N* = 3, *n* = 12. **(f)**, Quantification of hyperphosphorylated tau by western blot of Ser202 phosphorylated tau (CP13 antibody) with background subtracted and normalized to β-actin. **(g)**, Quantification of hyperphosphorylated tau expression by western blot of Ser202/Thr205 phosphorylated tau isoforms (AT8 antibody) with background subtracted and normalized to β-actin. **(h)**, Representative immunofluorescence images of the CA3, CA1, and CTX with NeuN (magenta) stain. Scale bar = 50μm. **i-k**, Quantification of NeuN by raw integrated density in the **(i)**, CA3, **(j)**, CA1, and **(k)**, CTX. *N* = 5, *n* = 10. *p<0.05, **p<0.01, ***p<0.005, ****p<0.001. Error bars = SEM. Statistical analysis was performed using two-way ANOVA with Fisher’s LSD **(b-d, f, g)**. Statistical analysis done using two-way ANOVA with Tukey’s multiple comparisons test. **(i-k)**.

After assessing the effects of STM2457 on cognition, learning, and memory in PS19 mice, we examined brain tissue from 9-month-old mice to determine whether pathological tau accumulation or neurodegeneration had been alleviated. Immunofluorescent staining of brain sections revealed depleted levels of excessive MC1-positive misfolded tau in PS19 mice treated with STM2457 (Fig. 5a–d, **Extended Data Fig. 3a, b**). Changes in MC1 were also accompanied by changes in m^6^A demonstrated previously (Fig. 3g–i). This depletion was evident in the CTX, CA3, and CA1 (Fig. 5b–d). Hyperphosphorylation of tau in PS19 mice was assessed using the phosphorylation-specific antibodies CP13 and AT8, which recognize pathologically relevant tau phosphorylated at Ser202 and Ser202/Thr205, respectively. Western blot analysis showed that levels of both CP13- and AT8-positive tau were elevated in untreated PS19 mice compared to WT. STM2457 treatment significantly reduced tau phosphorylation at the Ser202 and Ser202/Thr205 sites in PS19 mice (Fig. 5e–g). Together, these data demonstrate that, alongside cognitive improvements, STM2457 treatment can reduce multiple forms of misfolded and hyperphosphorylated tau pathology.

Based on the reduction in tau pathology and improvement in cognitive behavior, we continued to investigate whether neurodegeneration was slowed or prevented. To assess neuronal integrity, PS19 brain tissue was stained with the NeuN antibody (a Neuronal Nuclei marker). Untreated PS19 mice showed reduced neuronal integrity in the CA1 and cortex relative to WT (Fig. 5h–k). Compared to untreated PS19 mice, STM2457-treated mice exhibited significantly increased neuronal density in the CA3, CA1, and cortex, restoring levels to those observed in WT mice (Fig. 5i–k).

In summation, these results demonstrate that STM2457 treatment mitigates tau pathology and protect neurons against degeneration in brain regions bearing pathological tau aggregation and excessive m^6^A accumulation.

### STM2457 treatment attenuates glial inflammatory response in PS19 mice.

AD pathology is associated with neuroinflammation involving altered glial cell activation, including neuroprotective acute activation and neurotoxic chronic activation^45,46^. Additionally, m^6^A modification has been implicated in regulating microglial inflammatory responses and microglia-neuron interactions^20,47,48^. Considering the effects STM2457 had on cognition, tau pathology, and neurodegeneration, we next considered how glial activation states change with treatment in PS19 mice.

**Figure 6:**
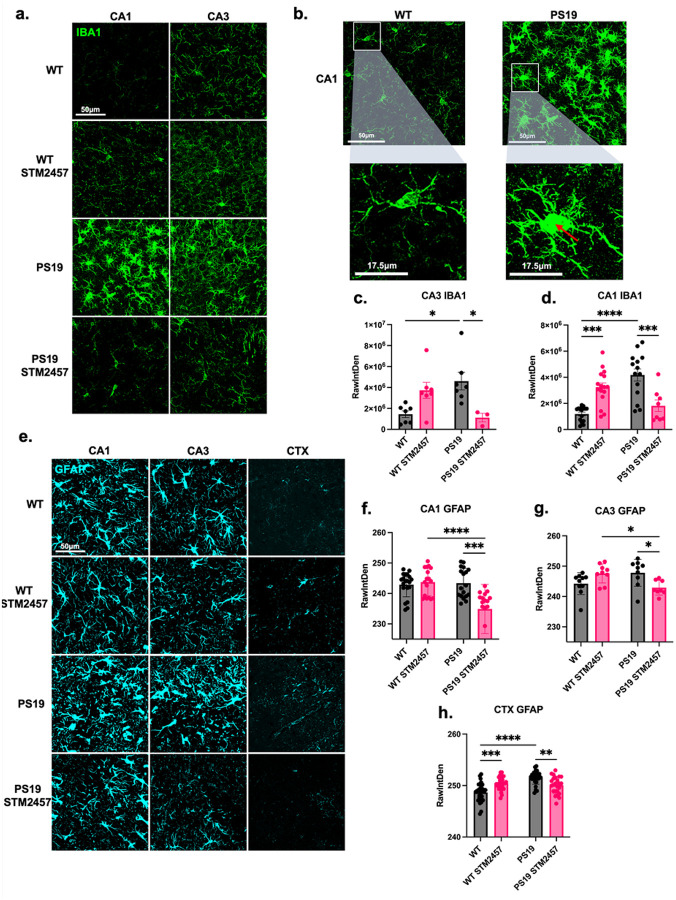
Treatment with STM2457 attenuates glial inflammatory response in PS19 mice. **(a)**, Representative immunofluorescence images of CA1 and CA3 of treated and control PS19 and WT mice with IBA1 (green). Scale bar = 50μm. **(b)**, Representative immunofluorescence image magnification illustrating resting state microglial morphology in WT CA1 (left) and ameboid-like morphology of inflammatory microglia cell body (red arrow) in PS19 CA1 (right). Scale bar = 17.5μm. **c, d**, Quantification of immunofluorescence intensity of IBA1 by raw integrated density (RawIntDen) in the **(c)**, CA1, and **(d)**, CA3. **(e)**, Representative immunofluorescence images of the CA1, CA3, and CTX of treated and control PS19 and WT mice with GFAP (cyan). Scale bar = 50μm. **f-h**, Quantification of GFAP immunofluorescent intensity by RawIntDen in the **(f)**, CA1, **(g)**, CA3, and **(h)**, CTX. *N* = 5 brains per condition, *n*= 10 sections per condition. *p<0.05, **p<0.01, ***p<0.005, ****p<0.001. Error bars = SEM. Statistical analysis was done using two-way ANOVA with Tukey’s multiple comparisons test.

To elucidate the impact of STM2457 on AD-related glial activation, we examined the morphological changes of microglia and astrocytes indicative of the shift into differently activated inflammatory states. We used an IBA1 antibody to mark both resting and activated microglia and used immunofluorescence intensity to assess morphological alteration and activation status. Immunofluorescent staining of hippocampal (CA1 and CA3) tissue from 9-month-old PS19 mice revealed increased ameboid-like morphology of microglia and associated increased fluorescence intensity that was attenuated in mice that received STM2457 treatment (Fig. 6a–d). Interestingly, STM2457-treated WT mice showed a slight increase in IBA1-positive microglial intensity, despite the absence of detectable pathology or behavioral changes. This may indicate a more proactive microglial state associated with METTL3 inhibition by STM2457, potentially reflecting its role in maintaining m^6^A homeostasis.

A similar investigation of astrogliosis-associated morphology revealed increased fluorescence intensity indicative of hypertrophy of the cell body and processes in the CA1, CA3, and CTX of 9-month-old PS19 mice; this reactive morphology was quelled by STM2457 treatment (Fig. 6e–h).

Altogether, these findings indicate that, in addition to reducing tau pathology and restoring neuronal integrity, STM2457 treatment also reduces the neuroinflammatory responses of microglia and astrocytes.

## Discussion

Epigenetic modifications are increasingly recognized as contributors to AD pathogenesis, highlighting new therapeutic opportunities. Among these, m^6^A RNA modification has emerged as a potential regulator of AD-related processes, though prior findings have varied across pathological contexts^19,30,49,50^. Our study supports a link between excessive m^6^A and tau pathology and shows that inhibiting the m^6^A writer METTL3 with small molecule STM2457 reduces tau deposition, mitigates neurodegeneration, and prevents cognitive decline in PS19 mice.

Our analysis of post-mortem human brain tissue revealed that increased m^6^A modification is present in the frontal cortex (Brodmann’s area 10) of AD brains, which is consistent with previous findings in the same brain region^30^. We also observed that m^6^A levels rise concurrently with increasing Braak stage, hyperphosphorylated tau accumulation, and Aβ plaque deposition. To be noted, other studies also reported inconsistent m^6^A alterations across cortical regions in human post-mortem brain tissue, including reduced m^6^A in the anterior cingulate gyrus and middle temporal gyrus^19,31^. These discrepancies suggest region-specific regulation of m^6^A and highlight the need for comprehensive spatial profiling of m^6^A in both diseased and non-diseased human brains.

Using humanized P301S tau transgenic mouse models of AD, we found that PS19 mice exhibit elevated m^6^A levels in the cortex and hippocampus at 9 months compared to age-matched WT and levels increased further from 6 to 9 months. These findings align with prior reports that m^6^A varies with age, both independently and in the context of aging-related diseases^20,31,36,51^. Given the prominence of tau pathology in the hippocampus and cortex, this increase suggests a potential age-related link between elevated m^6^A RNA and tau accumulation, highlighting the dynamic and transient nature of m^6^A regulation in the brain^5^.

Our findings in human AD cases and PS19 mice support the modulation of m^6^A levels by targeting its regulatory proteins, such as methyltransferase METTL3, as a promising therapeutic strategy for AD. STM2457, originally developed as a selective METTL3 inhibitor for hematologic malignancies, demonstrates several pharmacological properties that favor potential clinical development, including its small molecule structure, nanomolar potency, ability to cross the blood brain barrier, and capacity to reduce m^6^A levels *in vivo*^37^. Treatment of PS19 mice with STM2457, delivered continuously for 6 weeks via a surgically implanted osmotic pump subcutaneously with precise dose control, reduced m^6^A levels to a range more comparable to age-matched WT mice as shown by immunolabeling and RNA dot blot. Assessment of STM2457 treatment on tau deposition revealed a robust reduction in MC1-positive misfolded tau, pTau (Ser202), and pTau (Ser202/Thr205) in the hippocampus and cortex of treated PS19 mice, indicating that removal of excessive m^6^A effectively limits pathological tau accumulation. Protein levels of key m^6^A regulators were assessed to elucidate the regulatory mechanisms and coordination underlying m^6^A homeostasis. METTL3 and METTL14 protein levels were significantly reduced in STM2457-treated PS19 mice. In contrast, STM2457-treated WT mice showed no change in METTL3 and a slight increase in METTL14 relative to untreated WT. This variance in translational effect suggests the presence of a compensatory mechanism that attempts to restore the balance of the m^6^A regulatory axis. Evaluation of neuronal density further revealed that reduced tau pathology in treated PS19 mice is accompanied by protection against neurodegeneration. Together, these findings demonstrated that STM2457 successfully inhibited METTL3 and reduced m^6^A methylation, decreased tau pathology, and mitigated neurodegeneration.

Glial activation is a key component of the inflammatory response to pathogenic tau and Aβ in AD^52–56^, and increasing evidence indicates that m^6^A RNA methylation participates in regulating neuroinflammatory cascades^48,57–61^. Recent studies have also shown differential clustering of m^6^A regulators in infiltrating immune cells from AD patients^62^, further suggesting a link between m^6^A signaling and immune activation. In this study we found that STM2457 treatment reduced microglial activation and astrogliosis in PS19 mice, as reflected by decreased inflammatory glial morphology. However, because glial activation in tauopathy may arise secondary to pathological tau aggregation, the reduction in inflammatory morphology could be partly attributable to decreased tau deposition. Even so, our findings also raise the possibility that m^6^A directly modulates glial immune responses. To more comprehensively evaluate this potential role, future studies are planned to assess additional inflammatory endpoints, including reactive oxygen species production and pro-inflammatory cytokine release.

Importantly, the therapeutic relevance of STM2457 lies in its ability to reverse cognitive impairment, one of the most debilitating features of AD. In our study, behavioral testing demonstrated that STM2457 fully rescued the learning, memory, and general cognitive deficits observed in PS19 mice. Improvements in locomotion in the open field test indicates that STM2457 mitigates motor impairments associated with this tauopathy model. These findings align with other studies reporting that reducing or eliminating METTL3 activity can benefit cognition in amyloid related AD mouse models^63,64^. Notably, unlike previous work that has focused predominantly on amyloid-based models, our study reveals a direct role for m^6^A in modulating tau pathology, which is particularly important because tau burden shows a stronger correlation with cognitive decline in AD^65^. This distinction underscores the unique contribution of our findings in supporting the role of m^6^A as a key regulator of tau-driven neurodegeneration.

Taken together with prior studies, our findings support the therapeutic potential of targeting m^6^A in AD. m^6^A RNA modification influences multiple disease-relevant pathways, including tau misfolding, Aβ clearance, neuroinflammation, and synaptic function^19,36,47,49,66^. Regional and stage-dependent variation in m^6^A patterns further indicate that modulating METTL3 could restore disrupted transcriptomic regulation in a context-specific manner. Specifically, the prolonged presence of STM2457 in the hippocampus following sustained subcutaneous delivery via osmotic pumps enhances its potential as a viable therapeutic agent. Nevertheless, m^6^A is regulated by complex enzymatic networks, and their remain questions regarding the consequences of long-term METTL3 inhibition. Our observation that METTL3 and METTL14 respond differently to STM2457 in WT versus PS19 mice also points to distinct compensatory mechanisms that require deeper investigation. Overall, selective METTL3 inhibitors represent a promising therapeutic avenue, and advancing STM2457 and related compounds will require continued evaluation of safety, dosing, and cellular consequences to support future clinical translation.

In conclusion, this work suggests that reducing excessive m^6^A RNA levels using STM2457 reduces tau pathology, neurodegeneration, neuroinflammatory glial morphology, and improves learning and memory. This impact of m^6^A methylation on tau pathogenesis presents new avenues for therapeutic intervention. This work highlights the potential of STM2457 as a novel AD therapeutic, whose impact on m^6^A methylation in specific cell types and pathogenic processes needs further investigation.

## Materials and Methods

### Human post-mortem brain tissue

Anonymous human brain tissue used in this project was obtained from the Goizuetta Alzheimer’s Disease Center and was collected in accordance with IRB protocols of Emory University. Human anterior prefrontal cortex (Brodmann Area 10) was used for immunohistochemical analysis and immunofluorescence labeling in the current study. These brain regions were selected based on consistent evidence from prior studies indicating a strong positive correlation between altered gene expression and disease severity^67^. The samples were de-identified and are described below. The tissue was fixed in periodate-lysine-paraformaldehyde (PLP) fixative for 2 hrs, followed by overnight incubation in 30% sucrose, after which tissue sections were cut at thickness of 30μm.

### Fixed human brain samples

The human brain tissue samples used in this study were all de-identified. All studies included both sexes, and results were integrated by covariate analysis, as described below.

**Table:** 

Primary Neuropathologic Diagnosis	Braak Stage	Age at Onset	Age at Death/Bx	Duration (years)	ApoE	Race	Sex
Control	I	-	59	-	E2/3	b	m
Control	I	-	70	-	E3/3	b	m
Control	I	-	72	-	E3/3	w	m
Control	II	-	78	-	E3/3	w	f
Control	IV	-	84	-	E2/3	b	f
Control	III	-	91	-	E3/3	w	f
Control	III	-	92	-	E3/3	w	f
Control	II	-	94	-	E3/3	w	m
Control/asymptomatic AD	II	-	64	-	E4/4	w	f
Control/asymptomatic AD	IV	-	80	-	E3/4	w	m
Control/asymptomatic AD	II	-	81	-	E3/3	w	m
Control/asymptomatic AD	I	-	87	-	E3/4	w	m
Control/asymptomatic AD	III	-	87	-	E2/3	w	f
Control/asymptomatic AD	IV	-	89	-	E3/3	w	m
Control/asymptomatic AD	IV	-	91	-	E3/3	w	m
AD	VI	49	59	10	E3/3	w	m
AD	VI	59	69	10	E3/4	w	m
AD	VI	53	71	18	E3/4	w	m
AD	VI	65	80	15	E3/4	b	f
AD	V	77	83	6	NA	w	f
AD	V	86	92	6	E3/4	w	f
AD	V	82	92	10	E3/3	w	m
AD	VI	80	93	13	E3/4	w	f

### Animals

Use of all animals was approved by the University of Virginia Institutional and Animal Care and Use Committee. All animals were housed in an IACUC-approved vivarium at the University of Virginia School of Medicine. P301S tau PS19 transgenic mice: PS19 mice overexpressing human P301S Tau (B6;C3-Tg(Prnp-MAPT*P301S) PS19Vle/J, stock #008169) were purchased from Jackson Laboratories. Male PS19 P301S tau^+/−^ mice and female C57BL/6 wild type were used as breeding pairs and the F1 generation of P301S tau^+/−^(PS19) and P301S tau^−/−^ (wild type) were used for the experiment. Littermates of the same sex were randomly assigned to experimental groups. Mice were sacrificed for experimentation at the age of 6- and 9-months-old, respectively.

The perfusion and fixation method used for brain tissue was critical for successful immunostaining conducted in this study. Following anesthesia, mice were perfused through the heart with 20mL ice cold PBS at the speed of 4mL/min for 10 mins until the mouse tail became curved and stiff. The mouse brains were dissected and placed in 4% paraformaldehyde (PFA) on ice for 24 hrs before being transferred 0.05% sodium azide/PBS solution. To prepare for brain sectioning, the fixed mice brains were transferred into 30% sucrose/PBS until the brains sank to the bottom of the tube (about 48h) and then sectioned. The fixed brains were cryosectioned into 30μm coronal sections and stored in 0.005% sodium azide/PBS solution at 4°C for up to 3 months. For long-term storage, the sections were transferred into cryoprotectant solution (30% glycerol and 30% ethylene glycol in PBS) and stored at −20 °C.

### Immunohistochemistry staining

Floating 30μm brain sections were placed onto slides. All subsequent steps were performed at room temperature unless otherwise specified. Sections were outlined with a hydrophobic barrier using a PAP pen and air-dried before staining. Sections were washed in PBS for 10 min then treated with 1% H2O2 diluted in dH2O for 15 min. Sections were rinsed twice in PBS for 15 min each, then blocked with 0.4% Triton X-100 with 1% Bovine serum albumin (BSA) and 4% normal goat serum (NGS) in PBS for 30 min. After blocking, sections were incubated overnight in 1° antibody diluted in DAKO antibody diluent (5% BSA in 0.25% TritonX-100/PBS) at 4°C with photobleaching. The following day, sections were rinsed twice in PBS for 15 min then incubated for one hr in BTA solution (0.44% Biotinylated goat anti-mouse IgG diluted in 0.3% Triton X-100/PBS). Sections were rinsed twice in PBS then treated with Avidin Biotin Complex (ABC) (0.88% Avidin, 0.88% Biotin diluted in 0.3% Triton X-100/PBS, prepared 30 min prior to use; Vector Laboratories, Cat# PK-6101). After ABC incubation, sections were washed three times in PBS for 10 min each. DAB solution was prepared by adding 1 tablet DAB (Millipore Sigma, cat# D4293–5SET) and 1 tablet Urea (Sigma-Aldrich, Cat# D4193) to 5mL dH2O and vortexed until fully dissolved. DAB solution was added to sections individually for one to three min until section turned medium brown in color, then were immediately placed in PBS. Sections were washed twice with PBS for five min each, then dried overnight at 37°C. The following day, sections were dehydrated by washing in 70% EtOH, 85% EtOH, 95% EtOH, and 100% EtOH for two min each followed by washing in 100% EtOH for five min. Slides were then cleared by washing twice in Xylene for five min followed by immediately dropping 50μL of Permount Mounting Medium (Thermo Fisher Scientific, Cat# SP15–500) and covering with glass coverslips. Slides were dried overnight in the fume hood and stored at 4°C for long term storage. All washing and incubated steps were done with agitation. Primary antibody used for brain tissue DAB staining was mouse monoclonal purified IgG anti-m^6^A, 1:2000 (Synaptic Systems, Cat# 202 011, RRID: AB_2619890).

### RNA dot blot

RNA dot blot protocol was adapted from Zhao et al.^64^ RNA extraction was performed using RNeasy Plus Universal Mini Kit (73404, Qiagen) on ~20–25mg of cortical tissue that had been frozen in RNA later. ~400ng/μL of extracted RNA was heated to 95°C for 3 min to disrupt secondary structures, then samples were immediately placed on ice. Then 1μL 10mM HEPES buffer and 1μL methanol were added to the RNA, and the volume was brought up to 10μL using molecular biology grade water. Then 10μL of each sample was dotted onto a positively charged nylon membrane (Cytiva, Amersham Hybond -N+, RPN1210B). Following this, the membrane was heated in an oven to 65°C for 45 min, then immediately placed in a UV crosslinker with the following settings: 125 mJoule/cm2 at 254 nM for 5 min. Then the membrane was washed with PBST (1X PBS, 0.01% Tween-20) with gentle shaking for 10 min. The membrane was then blocked in 5% skimmed milk powder in 1X PBS for 1 hr with gentle shaking at RT. The primary antibody used was mouse IgG3 anti-m6A monoclonal antibody (Proteintech, Cat # 68055–1-Ig, RRID: AB_2918796) in a 1:1000 dilution in 5% BSA in PBST (1X PBS, 0.01% Tween-20) solution. The membrane was submerged in primary antibody and incubated either at RT with gentle shaking for 2 hrs, or at 4°C with gentle shaking overnight (14–16 hrs). The membrane was then washed 3x in PBST for 10 min. A horseradish peroxidase concentrate was used as a secondary antibody, specifically goat anti-mouse IgG (H+L) horseradish peroxidase-conjugated secondary antibody (Thermo Fisher Scientific, Cat# G-21040, RRID: AB_2536527) was used at a 1:2000 dilution in 5% BSA in PBST. The membrane was incubated for 1 hr at RT with gentle shaking. The membrane was washed 4x for 10 min each in PBST with gentle shaking. Pierce^™^ ECL Western Blotting Substrate (Thermo Fisher Scientific, Cat #32106) was used for luminescent development: 4 mL of detection reagent 1 peroxidase solution and 4 mL of detection reagent 2 luminol enhancer were added to the membrane to incubate for 5 min. The membrane was then imaged on a BioRad ChemiDoc imaging system. Afterwards, the membrane was briefly washed with PBST to remove the ECL substrate and was then submerged in Pierce^™^ Restore Plus Western Blot Stripping Buffer (Thermo Fisher Scientific, Cat# 46428) for 5 min. Then the membrane is placed in a Methylene Blue staining buffer (0.2% methylene blue in 0.4M sodium acetate and 0.4M acetic acid) for 30 min with gentle shaking. The membrane was washed with molecular biology grade water until the solution was clear (~3 4–5-minute washes). Membrane was imaged on BioRad ChemiDoc imaging system using blue and green StarBright B700 and B520 Blot settings, respectively.

### Western blot

At the time of perfusion cortical tissue samples intended for western blot were snap frozen and transferred to a 1.5mL Protein LoBind Eppendorf tube and stored at −80°C. Samples were subsequently lysed by mechanical homogenization 40μL Hsiao TBS buffer (50mM Tris, pH 8.0, 274mM NaCl, 5mM KCl) supplemented with cOmplete Protease Inhibitor (Roche, Cat# 11836153001) and PhosSTOP Phosphatase Inhibitor (Roche, Cat# 04906845001). 200U of Benzonase (Sigma-Aldrich, cat# E1014–25KU) with 1mM MgCl2 was added for removal of nucleic acids. Protein lysate concentration was quantified by the Pierce BCA assay according to the manufacturer’s protocol (Thermo Fisher Scientific, cat# 23227). 10–20μg of protein lysate were separated by denaturing gel electrophoresis (SDS-PAGE) and transferred onto a nitrocellulose membrane. The membrane was blocked for an hr in 5% non-fat milk in .01% Tween-20 in 1x PBS (PBST), and incubated in primary antibody (dilution antibody dependent) in 5% BSA PBST overnight at 4°C. The following day, the membrane was washed with PBST for 3 times for 10 min each then incubated with secondary antibody goat anti-mouse IgG (H+L) horseradish peroxidase-conjugated secondary antibody 1:5000 (Thermo Fisher Scientific, Cat# G-21040, RRID: AB_2536527) in 5% BSA PBST for 2 hrs at RT. The membrane was washed with PBST 3 times for 10 min before briefly being washed with PBS prior to visualization. Protein bands were visualized using Pierce^™^ enhanced chemiluminescence (ECL) Western Blotting Substrate (Thermo Fisher Scientific, Cat# 32106) and imaged by ChemiDoc Imaging System (Bio-Rad). Band intensities were quantified using ImageJ. Protein abundance was normalized to total Beta Actin with background subtraction. Primary antibodies used for immunoblotting were as follows: : rabbit IgG METTL3 polyclonal antibody 1:2000 (Proteintech, Cat# 15073–1-AP, RRID: AB_2142033), rabbit IgG METTL14 polyclonal antibody 1:2000 (Proteintech, Cat# 26158–1-AP, RRID: AB_2800447), rabbit IgG ALKBH5 polyclonal antibody 1:1000 (Proteintech, Cat# 16837–1-AP, RRID: AB_2242665), mouse IgG Phospho-Tau (Ser202, Thr205) Moloclonal Antibody (AT8) 1:2000 (Invitrogen, Cat# MN1020, RRID: AB_223647); mouse anti-tau p202 (CP13) 1:500 (provided by Feinstein Institute through MTA) and mouse β-Actin Monoclonal Antibody 1:2000 (Proteintech, Cat# 66009–1-Ig, RRID: AB_2687938).

### Immunofluorescence labeling

Coronal sections, including cortex and hippocampus, were washed in PBS for 10 mins and then permeabilized in 0.5mL PBS/0.25% Triton X-100 (PBST). The selected brain tissues were then blocked in blocking solution (5% BSA and 5% normal donkey serum in PBST) for1.5–2 hrs at room temperature (RT). Then sections were incubated in primary antibodies diluted in 5% BSA/PBST overnight at 4°C. On the second day, brain sections were washed three times in PBST, 15 min each. Brain sections were then incubated in 2° antibodies (1:1000 for Alexa fluor-conjugated antibodies made in goat/donkey purchased from Thermo Fisher Scientific) diluted in 5% BSA/PBST for 2 hrs at room temperature. For DAPI nuclei stain, DAPI (1:10,000of 1mg/mL stock) was diluted in PBST and incubated with brain sections for 15 min followed by two washes with PBST then one wash with PBS, 10 min each. The brain sections were mounted onto microscope glass slides using Prolong gold antifade reagent. Images were captured by Leica Stellaris 5 confocal microscope. Primary antibodies used for brain tissue staining were as follows: mouse monoclonal purified IgG anti-m^6^A, 1:2000 (Synaptic Systems, Cat# 202 011, RRID: AB_2619890); mouse anti-MC1, 1:300 (provided by Feinstein Institute through MTA); chicken anti-microtubule-associated protein antibody, 1:1000 (Aves Labs, Cat# MAP, RRID: AB_2313549); rabbit anti-Iba1, 1:1000 (Fuji Film Wako Pure Chemical Corporation, Cat# 019–19741, RRID: AB_839504); rat monoclonal antibody anti-GFAP, 1:1000 (Thermo Fisher Scientific, Cat# 13–0300, RRID: AB_2532994); guinea pig polyclonal anti-NeuN antibody (Synaptic Systems, Cat# 266 004, RRID: AB_2619988).

### STM2457 treatment via osmotic pump delivery

STM2457 (MedChem Express, Cat#HY-134836), a potent and selective METTL3 inhibitor (IC_50_ = 16.9nM), is delivered via Alzet osmotic pump model 2006 for continuous administration (0.15μL/hr, 42-day duration, 200μL reservoir volume). Osmotic pumps are miniature infusion devices designed for controlled and continuous agent delivery in laboratory animals, commonly used for systemic administration when implanted subcutaneously.

In our experiment, the working solution of STM2457 at 2.08mg/mL (4.6mM) was prepared and immediately followed by 200μL infused into the osmotic pump to maintain accurate dosing at 0.25mg/kg/day *in vivo*. The osmotic pumps were then implanted subcutaneously on the neck of the 4-month-old mice (~30mg), including wild type control, and PS19 *(N=*10 mice per condition). This ensured precise and sustained release of STM2457 at a rate of 0.15μL/h (0.312μg/h) for 42 days.

For preparation of STM2457 working solution, it was first dissolved in DMSO at 50mg/mL (112.48mM) with ultrasonic treatment and heated to 60°C to ensure complete dissolution while avoiding repeated freeze-thaw cycles. The stock solution is stored at −80°C for up to 6 months or at −20°C for up to 1 month. The final working solution is prepared by sequentially adding 10% DMSO, 40% PEG300, 5% Tween-80, and 45% saline, yielding a clear solution with solubility ≥ 2.08mg/mL. This formulation ensures stability and consistent STM2457 delivery via subcutaneously implanted osmotic pump.

### Liquid chromatography–mass spectrometry (LC-MS)

10mg of Hippocampal frozen tissues were pestled, followed by addition of 200μL of ethyl alcohol water mixture (50% ethyl alcohol:50%water). The Tissue + solvent samples were vortexed for 10 min under ambient temperature. After vortex, Tissue + solvent samples were centrifuged at 10000 RPM for 10 mins. 170μL of supernatants were collected and aliquoted. The whole extraction process was repeated three times. 80μL of supernatants were used for non-spiked LC-MS analysis and 90μL of supernatants were used for spiked LC-MS analysis. 10μL of 116μM STM2457 standard solution were spiked into 90μL supernatants to reach a rough final concentration of 11.6μM.

Data-dependent acquisition (DDA) analyses were performed on Orbitrap Exploris coupled to Vanquish system (Thermo Fisher Scientific). 10μL of supernatants were separated on a HALO 90 Å AQ-C18 column (4.6 × 150 mm, 5μm, C18, Advanced Materials Technology, Delaware, Cat# USEQT001307) at 250μL per min. Mobile phase A consisted of 50% LC/MS grade H_2_O (W6–4, Fisher Scientific) and 50% LC/MS grade methanol (34860–4L-R, Sigma-Aldrich), mobile phase B consisted of LC/MS grade ethyl alcohol (459829–4L, Sigma-Aldrich), and both mobile phases contained 0.1% FA. The LC gradient was: 0% B to 50% B in 5 min, 98% B in 10 min until 12 min, and 0% B in 15 min, with a total gradient length of 15 min. A targeted MS1 scan and a ddMS2 scan were paired for quantification and qualification purpose. Targeted MS1spectra were collected at 120,000 resolution and ddMS2 spectra at 90,000 resolution. 70% HCD collision energy was used for ddMS2 fragmentation.

### Elevated plus maze

The elevated plus maze (EPM) protocol was adapted from Marco et al^68^. The EPM was used to assess cognition similar to the application in Jürgensen et al^43^. The apparatus consisted of two opposing closed arms covered by high walls (20 cm tall opaque black Plexiglass, 35 × 6 cm^2^), while the other two arms remained open to the air (uncovered, 35 × 6 cm^2^) extended from a common center square, elevated 121cm above the floor. Mice were placed onto the opaque white Plexiglass floor in the center facing a closed arm and allowed to explore the entire raised platform for 5 min. Data was recorded using an overhead camera with the EthoVision XT automated tracking system (Nodulus Information Technology), which provided the time spent in the open and closed arms. Between trials, the maze was thoroughly cleaned with 70% ethanol.

### Open field

The open field test protocol was adapted from Lammert et al.^69^ and was used to evaluate spontaneous locomotor activity. The open field consists of a square area (40 × 40 cm^2^) with white Plexiglass walls and floor, evenly illuminated with dim lighting. All mice were individually placed in the upper left corner of the open field and allowed to explore the area for 10 min. Movements were recorded using an overhead camera and data was collected using the EthoVision XT automated tracking system (Nodulus Information Technology). Locomotor activity was quantified as total distance traveled (cm). Between trials, the maze was thoroughly cleaned with 70% ethanol.

### Morris water maze

The Morris water maze (MWM) protocol was adapted from March et al^68^ and Vorhees & Williams^40^. The MWM was used to evaluate spatial learning and memory in 9-month-old mice WT and PS19 mice. The mice were placed in a 1m diameter pool of opaque water. The water was made opaque by adding a nontoxic tempera white paint and the water was kept at room temperature. A hidden 10-cm diameter platform was placed at approximately 1cm underneath the surface of the water on which the mice can stand on. The maze was divided into four quadrants. Four trials were performed each day across a four-day learning period. The mice were placed in the pool at various starting positions throughout the three quadrants that did not contain the platform. Four large markers (a square, circle, triangle, and X) were placed on the tub walls which provide visual cues to allow the mice to determine platform location. On Day-1 the mice were placed in their starting positions and allowed to search for the platform for 60s. Each mouse was allowed to remain on the platform for 2 min for the first trial, 1 minute the second trial, 30s the third trial, and 5s the last trial before being removed from the maze. If the mouse did not find the platform within 60s, the time to find the platform was recorded as 60s. Days 2–4 were all the exact same where the mice underwent four trials and had 60s to locate the platform. On these days the mice had to remain on the platform for 5s before being removed from the maze. Days 5 and 6 were “probe” days used to assess memory function, these days only contained single trials where the platform had been removed. The mice were once again allowed to search for the platform for 60s and the time spent in the quadrant where the platform was previously located was recorded. Day 7 of the test included four trials and had the platform returned to the same location as Days 1–4 and the mice were given 60s to locate the platform and had to remain on the platform for at least 5s before being removed from the maze. During the test data was recorded using an overhead camera and with the EthoVision XT automated tracking system (Nodulus Information Technology) which provided the average distance from the platform (cm), total swimming time, swimming time in the target vs. opposing quadrants, and the path taken to reach the platform. The time for the mice to reach the platform on Day 7 was considered escape latency. Between trials, the maze was thoroughly cleaned with 70% ethanol.

### Y-maze

The Y-maze protocol was adapted from Kraeuter et al^70^. The Y-maze was used to evaluate spatial working memory. The apparatus consisted of three identical opaque arms (35 cm long × 8 cm wide × 15 cm high) positioned at 120° angles, these arms were labeled A, B, and C. For this protocol we designated the arm labeled “A” as the starting arm, with the arms labeled “B” and “C” designated as non-starting arms. Each mouse was placed in the distal part of the starting arm of the maze, facing the center of the maze, and allowed to explore all arms freely for 8 min. An arm entry was defined as the center point of the mouse entering the arm. The number of spontaneous entries included the sum of the entries the mice made into the non-starting arms. The number of alterations included how many times a mouse entered all three arms consecutively (e.g., ABC, ACB). The total number of arm entries included the sum of all entries into any arm. The percent spontaneous alternation was calculated as:

%SpontaneousAlteration=(Numberofalterations)/[Totalnumberofarmentries-2])*100

Data was recorded using an overhead camera and with the EthoVision XT automated tracking system (Nodulus Information Technology). Between trials, the maze was thoroughly cleaned with 70% ethanol.

### Schematics

Schematics were created with BioRender.com

### Brightfield image analysis

Bright images (DAB) were captured by Keyence BZ-X810 Microscope (Keyence, BZ-X810) using BZ-X800 Viewer Software (Keyence). The staining intensities in DAB labeled brain sections were measured by ImageJ. Raw integrated density (RawIntDen) was recorded and normalized by area measured when appropriate.

### Fluorescent Image analysis

Fluorescent images were captured by STELLARIS 5 Confocal Microscope (Leica Microsystems, Stellaris 5) using LAS X Life Science Microscope Software Platform (Leica Microsystems). The staining intensities in immunofluorescence-labeled brain sections were measured by ImageJ. Either integrated density (IntDen) or raw integrated density (RawIntDen) were recorded and normalized by area measured when appropriate.

### Statistical analysis

Statistical analyses and figures artwork were performed using GraphPad Prism version 10.2.0 for Windows. Pairwise data was compared using unpaired student’s t-test. All group data are expressed as mean ± SEM. Column means were compared using one-way ANOVA. Group means were compared using two-way ANOVA with factors of genotype, age time course, or treatment status. When two-way ANOVA showed a significant difference, pairwise comparisons between group means were examined by Tukey’s or uncorrected Fisher’s LSD multiple comparisons test. Significance was defined at *p* < 0.05.

## Supplementary Material

Supplementary Files

This is a list of supplementary files associated with this preprint. Click to download.

- m6AMSSupplemental20251216.docx

## Data Availability

All data reported in this paper will be shared by the lead contact upon request. The raw IF, DAB and WB images, and the animal behavior test videos and analysis will be made publicly available at Mendeley Data (Reserved DOI:10.17632/pynkg2hdcf.1).

## Highlights

- N6-methyladenosine (m^6^A) is elevated in human AD brain tissue and correlates with both tau and Aβ pathology.
- m^6^A elevation is age dependent and closely correlated with pathological progression in a tauopathy mouse model of AD.
- METTL3 inhibition reduces m^6^A levels and alleviates tau pathology, neurodegeneration, and neuroinflammation in PS19 mice.
- STM2457 treatment improves learning and memory, supporting METTL3 as a promising therapeutic target for AD.
